# Syndromic Testing in the Pandemic Era and Beyond: Rapid Detection for Respiratory Infections in Istanbul

**DOI:** 10.3390/v17060776

**Published:** 2025-05-29

**Authors:** Mustafa Onel, Hayriye Kırkoyun Uysal, Arat Hulikyan, Yasemin Ayse Ucar, Gizem Yapar, Aytaj Allahverdiyeva, Serra Zeynep Akkoyunlu, Eray Yurtseven, Mehmet Demirci, Sevim Mese, Ali Agacfidan

**Affiliations:** 1 Department of Medical Microbiology, Istanbul Faculty of Medicine, Istanbul University, Istanbul 34093, Turkey; onelm@istanbul.edu.tr (M.O.); arathulikyan@hotmail.com (A.H.); dr.aytach92@gmail.com (A.A.); drsevimmese@gmail.com (S.M.); aagacfidan@hotmail.com (A.A.); 2 Department of Medical Microbiology, Faculty of Medicine, Istanbul Beykent University, Istanbul 34520, Turkey; yaseminayseucar@gmail.com; 3 Department of Medical Microbiology, Faculty of Medicine, Istanbul Aydın University, Istanbul 34295, Turkey; gizemyapaar@gmail.com; 4 Department of Medical Microbiology, Azerbaijan Medical University, Baku 370022, Azerbaijan; 5 Department of Biostatistics, Istanbul Faculty of Medicine, Istanbul University, Istanbul 34093, Turkey; serraakkoyunlu@gmail.com (S.Z.A.); eyurt@istanbul.edu.tr (E.Y.); 6 Department of Medical Microbiology, Faculty of Medicine, Kırklareli University, Kırklareli 39100, Turkey; mdemirci1979@gmail.com

**Keywords:** SARS-CoV-2, respiratory infection, respiratory viruses, QIAstat-Dx1 respiratory SARS-CoV-2 panel

## Abstract

The aim of the study was to determine the prevalence rates of respiratory pathogens using syndromic tests and also to show which respiratory viruses were detected in suspected cases, especially during and after the pandemic period. A total of 1984 different respiratory tract samples from various departments were included and studied with the QIAstat-Dx device in 2021–2023. The samples were studied with the QIAstat-Dx1 Respiratory SARS-CoV-2 Panel. The kit used was a fully automated, multiplex syndromic test that detected SARS-CoV-2 and 21 other respiratory tract pathogens. As a result of the study, the prevalence of Rhinovirus/Enterovirus (RV/EV) (18.59%), RV/EV-SARS-CoV-2 (42.74%), SARS-CoV-2 (5.04%), and Influenza A Virus (IAV) (5.59%) agents was found to be higher than other agents during the period investigated. Among the 1984 patients examined, 959 (48.33%) had a single viral agent, 156 (7.86%) had double coinfection, 11 (0.55%) had triple coinfection and 1 patient had quadruple coinfection. Nearly half of the patients had a straightforward infection, which helps clinicians in directing specific treatment methods. The study results demonstrate that during the pandemic period, the detection of respiratory pathogens such as SARS-CoV-2 and RV/EV was not only critical for accurate diagnosis but also served as an important indicator of the broader epidemiological trends in respiratory infections. The seasonal distribution showed that while RV/EV was frequently present, its coinfection with SARS-CoV-2 was notably observed only in the first trimester. In light of our findings showing high rates of SARS-CoV-2 and RV/EV detection, along with diverse patterns of coinfection in clinical samples, such comprehensive testing not only assists in rapid diagnosis but also informs public health strategies by reflecting the evolving landscape of respiratory infections in the pandemic and post-pandemic era.

## 1. Introduction

Respiratory tract infections (RTIs) affect the organs and tissues of the respiratory system, including the nose, throat, sinuses, larynx, trachea, bronchi, and lungs. These infections are most often caused by viruses or bacteria (or both). They are more likely to occur in those with weakened immune systems. They can cause death in this group. The elderly and children are the primary risk groups. RTIs are detected at all ages, especially in newborns and children, and continue to be an important public health problem, causing morbidity and mortality. Although it has been reported that viruses are responsible for 22–59% of infections, epidemiological data might show geographical and seasonal variations. In particular, viruses that cause RTIs can have serious effects because they can cause epidemics or pandemics all over the world [1,2,3]. The primary target of RTI viruses is airway epithelial cells. It is already known that infection is limited to the upper respiratory tract, but it can also progress to the lower respiratory tract and cause clinical conditions such as bronchiolitis or pneumonia. The balance between immune response and viral infection may vary according to age, host defense mechanisms, and characteristics such as viral load. In recent years, molecular methods that enable the simultaneous detection of many viruses that cause RTIs have been frequently employed in routine virology diagnostic laboratories. As well as obtaining important data for appropriate antiviral treatment, these studies also combat antibiotic resistance by reducing unnecessary antibiotic use [4,5].

Viruses that commonly cause RTI include rhinoviruses (RVs), respiratory syncytial virus (RSV), influenza and parainfluenza viruses (PIVs), coronaviruses, bocavirus, metapneumoviruses, adenoviruses and enteroviruses. RSV, influenza virus, and severe acute respiratory syndrome-coronavirus-2 (SARS-CoV-2) during the pandemic period were responsible for many deaths despite widespread implementation of preventive activities and vaccination programs worldwide [6,7]. During the pandemic period, detection of other respiratory viruses in patients did not exclude SARS-CoV-2 co-infection and lower respiratory tract (LRT) samples increased sensitivity in the diagnosis of viral respiratory tract infections, including SARS-CoV-2 [8,9]. Screening programs conducted starting in 2019 showed that seasonal human coronavirus and rhinovirus/enterovirus (RV/EV) positivity before the pandemic period was mostly replaced by SARS-CoV-2 during the pandemic period [10].

In the present day, after the development of new diagnostic tests, clinicians can diagnose patients faster and apply appropriate treatment. Therefore, it has become easier to monitor the patient’s condition and clinical course. With the developed multiplex PCR tests, it is possible to reliably detect many viral agents in the same clinical sample. During the COVID-19 pandemic period, the diagnostic tests used have become a fundamental tool in the fight against the spread of SARS-CoV-2 and have attracted great attention. During this period, while societies around the world were vaccinated at a high rate, it was seen that rapid COVID-19 tests played an important role in the recovery of the economy. Since the beginning of the pandemic, many diagnostic devices have been developed and launched for routine testing [11,12]. The aim of the study was to determine the prevalence rates of respiratory pathogens using syndromic tests and also to show which respiratory viruses were detected in suspected cases, especially during and after the pandemic period.

## 2. Materials and Methods

The patients included in the study were all patients with complaints of acute respiratory tract infection (fever, cough, respiratory distress, etc.) admitted to the Triage department of Istanbul Medical Faculty Hospital. In all these suspected patients, samples were collected consecutively without applying any exclusion criteria. Nasopharyngeal swab samples of 1984 patients were transported in a universal transport medium (Copan Diagnostic, Murrieta, CA, USA) to the laboratory of the department of medical microbiology, virology and basic immunology, Istanbul University, Istanbul Faculty of Medicine. After the samples were anonymized, a study number was assigned depending on patient demographic information including age, gender, and hospitalization status (general practice, hospitalized, pediatric hospital/ward, intensive care unit). Identification of viral agents from clinical samples was performed using the “QIAstat-Dx (Qiagen, Hilden, Germany) Respiratory SARS-CoV-2 Panel” on a fully automated, multiplex syndromic QIAstat-Dx analyzer (Qiagen, Hilden, Germany) in line with the manufacturer’s recommendations. This multiplex real-time polymerase chain reaction (Rt-PCR)-based test can simultaneously identify 22 different agents (Influenza A (IAV), IAV subtype H1N1/2009 (IAV-H1N1pdm2009), IAV subtype H1, IAV subtype H3, Influenza B (IB V), Coronavirus 229e, Coronavirus HKU1, Coronavirus NL63, Coronavirus OC43, PIV 1, PIV 2, PIV 3, PIV 4, RSV A/B, Human Metapneumovirus A/B (hMPV), Human Adenovirus (hAV), Human Bocavirus (hBoV), Rhinovirus/Enterovirus (RV/EV), SARS-CoV-2, Mycoplasma Pneumoniae, Legionella Pneumophila, and Bordetella Pertussis). JASP (0.19.3) software and IBM SPSS Statistics V.30 program were used for statistical analyses.

## 3. Results

The study was conducted with 1984 nasopharyngeal swab samples in the department of medical microbiology, Istanbul Faculty of Medicine. Samples were collected consecutively from patients with complaints of acute respiratory tract infection (fever, cough, respiratory distress, etc.) admitted to the Triage department of Istanbul Medical Faculty Hospital and transported to microbiology laboratory.

The distribution of viral agents in four age groups (0–5, 6–18, 19–45, and >46) according to seasons in the 2021–2023 period was examined (in 3-month periods). Based on the results, in 1360 patients in the 0–5 age group, the following positivity rates were found: 21.03% RV/EV infection (*n* = 286; 117 females, 165 males, average 2.35), 10.44% RSV A/B (*n* = 142; 49 females, 92 males, average 0.79) and 3.82% SARS-CoV-2 (*n* = 52; 23 females, 29 males, average 0.56) positivity.

Similarly, 14.37% RV/EV positivity (*n* = 71; 30 females, 41 males, mean 9.76) was detected in 494 patients in the 6–18 age group. The second agent in the age group, IAV, was found to be 11.13% (*n* = 55; 17 females, 38 males, mean 9.79). In addition, 22.6% SARS-CoV-2 (*n* = 15; 9 female, 6 male, mean 30.4) and 13.24% RV/EV (*n* = 9; 8 females, 1 male, mean 27.2) positivity was found in 68 patients in the 19–45 age group. A total of 62 patients in the 46+ age group had 8.06% SARS-CoV-2 positivity (*n* = 5; 2 females, 3 males, mean 67.2), and 4.84% RSV, RV/EV and IAV.

In the present study, a single viral agent was detected in 959 patients, double coinfection in 156 patients, triple coinfection in 11 patients, and quadruple coinfection in 1 patient. Infection agents in the observed period mostly consisted of upper respiratory tract agents. Based on the obtained data, it was observed that RV/EV infection was quite common among patients applying to our hospital and its prevalence could indicate a projection for Istanbul province. The percentage distribution of the agents detected in the study according to age groups is shown in Table 1.

RV/EV in 2021, SARS-CoV-2 in 2022 and RV/EV in 2023 were the most frequently detected pathogens. Distributions of detected pathogens by years are shown in Table 2.

When three-month periods were taken into account, except for RV/EV-SARS-CoV-2 coinfection detected in period 1 (first trimester), RV/EV infection was frequently detected in other periods (Table 3).

When the monthly data of the pathogens were examined in the present study, it was determined that the *p*-values of the results were significant. The “N” symbol in Table 4 represents the 75 different infection combinations identified in the present study (Table 4).

Descriptive statistics were made for 75 different pathogen combinations identified and grouped from nasopharyngeal swab samples taken from 1984 patients and the distribution of pathogens among age groups was obtained. A t-test was used to determine whether there was a statistically significant difference between age groups for agents, and a significant difference was found between age groups (*p* < 0.001–0.013). Then, a proportional comparison of the detected pathogens was made according to gender.

These rates were made both for all patients without age difference and separately for four different age groups. Positive data and total negative data collected for each of the 75 different infection types for each month and for 3-month periods were recorded as percentage values. The data were examined on a monthly and agent basis. Parametric t-tests were performed on agent data that were compatible with normal distribution. Nonparametric tests were applied to agent data that were not normally distributed. The *p*-values obtained from these tests indicate that there is a statistically significant difference for each month (*p* < 0.001–0.006) and for 3-month periods (*p* < 0.001). Then, these factors were examined monthly and for 3-month periods. Similarly, parametric t-tests were performed on data with normal distribution. Factor data without normal distribution were examined with nonparametric tests. A significant change was observed for 13 factors monthly and a statistically significant difference was observed for two factors in 3-month periods.

## 4. Discussion

RTI is considered one of the important healthcare concerns on a global scale. It has a higher incidence, especially in children and some immunocompromised individuals, and symptoms ranging from cold to pneumonia are reported. Multiplex PCR tests are frequently used in laboratories in the routine diagnosis of RTI today. Infectious respiratory pathogens such as influenza, RSV, PIV, hMPV, RV, Influenza H3N2, COV, hAV and hBoV show different epidemiological distributions due to geographical variability or seasonality [13,14]. However, environmental factors, the development status of countries, economic conditions, and hygiene factors have caused the pathogen spectrum to change over the years. Early diagnosis of RTI agents helps clinicians to take preventive measures, which are the goal of reducing antibiotic misuse, shortening the duration of treatment, and increasing the effectiveness of treatment [15,16,17,18]. For these reasons, the study aimed to determine the prevalence rates of respiratory pathogens using syndromic testing methods. Significant variations in the prevalence of respiratory pathogens were observed and the positivity rates for RV/EV and SARS-CoV-2 were notably high during the pandemic period, followed by Influenza A Virus (IAV). In our results, we observed a seasonal pattern in the prevalence of these viruses, with specific increases in certain viruses like human RV/EV, RSV, and PIV3 during the spring and summer of 2021.

Early and accurate detection of the causative viruses is of great importance in determining the infections that occur as there are few clinical symptoms specific to RTI. Data on the regional distribution of respiratory viruses are necessary not only for local prevention and control of RTI but also for determining global health practices. In terms of treatment planning for the determined agents and implementation of agent-specific protection methods, RT-PCR tests with proven validity are preferred in routine laboratories today. Determination of RTI with high sensitivity and specificity by RT-PCR method provides early and accurate diagnosis of infections caused by these agents. The Multiplex PCR Method is used widely for the detection of many viruses in clinical samples today [19]. QIAstat-Dx analyzer (Qiagen, Hilden, Germany) was used in the present study. In total, 1124 positive cases were detected in 1984 samples by RT-PCR.

Based on the data from the WHO, CDC, and ECDC between 2021 and 2024, it was reported that the causative viruses other than COVID-19 in the positivity rate tests during the pandemic period were frequently RSV and influenza. However, it has been accepted by the scientific community that the incidence of RSV and influenza decreased due to the use of masks and compliance with social distancing rules in society during this period when the COVID-19 positivity rate was dominant [20]. RSV has a very high rate of infections as an RTI agent worldwide because it is generally effective in young children and older adults. Four types of influenza viruses (A, B, C, D) maintain their relevance throughout the year by causing seasonal epidemics. Although current vaccine treatments affect the immunization of society, the number of cases is increasing worldwide. Related symptoms are described as similar to those associated with the common cold. It also causes active infection in every age group. It might also cause serious consequences such as death if treatment is delayed in young children and older adults.

RSV is a leading cause of hospitalization for acute lower respiratory tract infection, especially in infants and young children. Current options for the prevention and treatment of RSV (Palivizumab) are limited to certain populations with high socioeconomic status. Several vaccines targeting young children, older adults, and pregnant women are currently being tested, and an effective and it is likely that a safe vaccine will become available soon [21].

In a previous study that was conducted by Petrocelli et al. on a total of 500 pediatric patients between 2015 and 2022, they found an increase in SARS-CoV-2 and RSV infections in newborns, especially in October and November 2021. The study was divided into three sample periods: pre-pandemic (2015–February 2020), pandemic period (quarantine period: March 2020) and post-pandemic (April 2022–November 2022). Positive cases diagnosed with RSV demonstrate that the virus is a pathogen that causes seasonal winter epidemics. Starting from 2015, it was observed that there was an increase in cases recorded for RSV in the winter season from November, peaked in January, and decreased in the spring/summer months. The seasonal trend of RSV cases seems to be stable in the pre-pandemic years. On the other hand, no cases were recorded from November 2020 to February 2021, which is considered to be due to the meticulous implementation of protection methods used in the fight against COVID-19 and other factors being ignored. Starting from September 2021, RSV and SARS-CoV-2 infection increased in newborns and children up to 11 years of age. Starting from January 2022, there has been a steady decrease in the number of RTIs caused by RSV in children. In September 2022, unlike 2021, no abnormal increases in RSV cases were observed [22]. In the present study, 157 RSV (7.86%) and 100 SARS-CoV-2 (5.04%) positivity rates were detected in a total of 1984 patients between 2021 and 2023.

Influenza is a seasonal contagious respiratory disease caused by the influenza virus that changes every year. For this reason, it is important to get vaccinated against influenza every year. For most people, it has mild symptoms similar to the common cold but might cause hospitalization and death among susceptible individuals (immunosuppressed patients, those with chronic diseases, and those aged 65+). Healthcare employees are at higher risk of infection due to the nature of their work. Globally, approximately 3–5 million people are known to be affected by severe influenza each year, and approximately 650,000 people die from the disease. Approximately 70,000 of these deaths occur in the WHO European Office region. Also, influenza can increase the risk of heart and circulatory problems such as heart attack and stroke [23].

A study by Moeren et al. was conducted in two hospitals in the Netherlands between October 31, 2022 and March 31, 2023. This study included patients aged 18–98 years, totalling 1740 patients, 1296 of whom (74.5%) were hospitalized. The female/male rate of these patients was reported to be 48.8%. Of the patients, 221 were positive for SARS-CoV-2, 148 for IAV, and 23 for IBV, corresponding to a prevalence of 12.7%, 8.5%, and 1.3%, respectively [24]. In the present study, 111 IAV (5.59%) and 11 IBV (0.55%) positivity rates were detected in 1984 patients.

In Ital, De Francesco et al. conducted a study on respiratory viruses and SARS-CoV-2 in hospitalized patients over the age of 18 in two periods: the pre-pandemic (January 2017–February 2020) and pandemic period (March 2020–May 2021). In this study, the test positivity/total patient number rates in the pre-pandemic period (January 2017–February 2020) were as follows: IAV 523/6881 (7.6%), IBV 210/6881 (3%), RSV 238/3240 (7.3%), hMPV 38/3240 (1.1%), hADV 48/3240 (1.4%), PIV (1,2,3,4) viruses 131/3240 (4%), RV 123/3240 (3.8%) and coronaviruses 164/3240 (5%). During the pandemic period (March 2020–May 2021), IAV 3/1628 (%0.18), IBV 1/1628 (%0.06), RSV 10/734 (%1.4), hMPV 2/734 (%0.27), hADV 4/734 (%0.6), parainfluenza viruses 1/734 (%0.14), RV 41/734 (%5.6) and coronaviruses 1/734 (%0.14) were detected. In addition, coinfections were also detected. Accordingly, in the pre-pandemic period, RSV was detected as the most common virus in coinfections (7/12, 58.3%). The only coinfection observed during the pandemic period was detected between RV and SARS-CoV-2 (six cases) [25]. In the present study, the following positivity rates were detected in 1984 patients: 369 RV/EV, 157 RSV A/B, 111 IAV, 100 SARS-COV-2, 43 hMPV, 41 hADV, 16 PIV4, 11 PIV3, 8 PIV2 and 1 PIV1 positivity.

In a previous study conducted by Li et al. in China with 160.436 samples, when the factors were examined in terms of co-infections, an increase in the prevalence of hMPV/RV and RV/AdV co-infections was found compared to other co-infections [26]. The current study’s findings align with previous research, such as the study by Li et al., which reported increased co-infections of hMPV-RV and RV-AdV during the pandemic period. However, our study found a lower rate of these specific co-infections, suggesting regional variations in viral interactions and the impact of public health measures like mask-wearing and social distancing. Additionally, while previous studies noted a decrease in RSV and influenza due to pandemic measures, our data highlight the persistent high positivity rates of RV/EV and SARS-CoV-2, underscoring the need for continued surveillance and adaptable diagnostic strategies in the post-pandemic era.

In a previous study conducted in Japan between 1 December 2020 and 31 March 2022, Kitagawa et al. examined 3177 patients with cold-like symptoms or respiratory symptoms. At least one virus was detected in 1215 of 3177 patients (38.2%), and a total of 1641 people were infected with the virus. Among these, human RE/EV (*n* = 655), SARS-CoV-2 (*n* = 264), RSV (*n* = 245), PIV3 (*n* = 193), seasonal coronaviruses (*n* = 131), hADV (*n* = 109), PIV4 (*n* = 36), PIV1 (*n* = 6), and PIV2 (*n* = 2) were detected. Influenza, PIV1, hMPV, M. Pneumoniae, Chlamydia Pneumoniae, B. Pertussis, or Bordetella Parapertussis were not detected during the study period. Among the 225 cases of SARS-CoV-2 infection, 23 cases of co-infection were encountered, including human RE/EV [*n* = 9], seasonal coronaviruses [*n* = 5], hADV [*n* = 1], human RE/EV and RSV [*n* = 4], human RE/EV and seasonal coronaviruses [*n* = 1], human RE/EV and PIV3 [*n* = 1], seasonal coronaviruses and PIV3 [*n* = 1], and seasonal coronaviruses and RSV [*n* = 1]. In this study, the number of detected viruses was found to be relatively low in the winter months from December 2020 to March 2021, followed by an increase in human RE/EV, RSV, and PIV3 in the spring and summer of 2021. After the summer of 2021, the number of human RE/EVs remained relatively high, while the number of other viruses detected was low after September 2021. They reported a rapid increase in the number of SARS-CoV-2 after December 2021 [27]. Our study in Istanbul similarly identified high positivity rates for RVEV and SARS-CoV-2, indicating a consistent pattern of these viruses being prevalent in different geographical regions during the pandemic period.

In a study by AlBahrani et al. using the QiaStat RT-PCR method in 1790 patients between January 2022 and November 2023, the most common were Rhinovirus/Enterovirus 222 (12.4%), RSV A/B 103 (5.7%), Influenza H1N1 77 (4.3%), Influenza A/B 172 (9.6%) and parainfluenza 58 (3.2%). SARS-CoV-2 was detected in 3.97% of the samples. The researchers determined fluctuations in the monthly prevalence of the pathogens identified over the two years. In the present study, including patients with coinfection in almost the same periods, RV/EV was 479 (24.14%), IAV/IBV 141 (7.10%), and SARS-CoV-2 120 (6.04%), respectively. There is a correlation between our results and literature data [28]. Our study supports this observation, as the high rates of RV/EV and SARS-CoV-2 detected during the pandemic period reflect the complex interplay between viral transmission and public health interventions. This connection highlights the need for continuous monitoring and adaptable diagnostic approaches to effectively manage respiratory infections in diverse settings.

In a study by Gosert et al. using the QiaStat RT-PCR method on 269 patients between November 2023 and February 2024, the most common cases were IAV/IBV 43 (15.98%), SARS-CoV-2 36 (13.38%), RSV 28 (10.40%) and hMPV 11 (4.08%), respectively. In the present study, hMPV was detected in 61 (3.07%) positive cases, including coinfections [29].

In another study conducted by Yılmaz et al. in Istanbul in the fall–winter seasons of 2021–2022, nasopharyngeal swab samples were taken from a total of 400 people (mean age 7.91 ± 17.80). The samples were evaluated with RT-PCR and multiplex PCR methods. When the virus distribution was examined, no significant difference was observed in the fall and winter months for RSV, COVID-19, and RE/EV, while hMPV, hADV, and IAV were found to be at higher rates in the winter months. PIV 1,2,3,4 and Coronavirus OC43 were detected at higher rates in the fall when compared to other viruses. Double and triple coinfection rates with other viral agents were found to be high for those aged two and under. The risk of coinfection of COVID-19 with IAV, RSV, PIV, and RE/EV was found to be higher than other viral agents. It was found that the risk of coinfection with IAV and COVID-19 increased especially in the winter months [30]. In the present study, 959 mono-agents, 156 double coinfections, 11 triple coinfections, and 1 quadruple coinfection were detected.

The use of data from a single institution located in Istanbul was one limitation of our study. In addition, the SARS-CoV-2 singleplex test result was not included in our study, which represents a second limitation. Our data were collected between the pre-COVID-19, pandemic and post-COVID-19 periods on the detection of SARS-CoV-2, especially during the pandemic period, but we also used singleplex tests in our laboratory. Nevertheless, our data are important in showing that syndromic tests can be an important tool in the follow-up of these pathogens, can quickly show us the status of these pathogens and are important for the detection of co-infections.

## 5. Conclusions

As a result of the examination of 1984 viral respiratory tract samples using syndromic testing between 2021 and 2023, this study observed a seasonal pattern in the prevalence of these viruses, with specific increases in certain viruses like human RV/EV, RSV, and PIV3 during the spring and summer of 2021. The positivity rates for RV/EV and SARS-CoV-2 were notably high during the pandemic period, followed by IAV. The implementation of COVID-19 protection methods, such as mask-wearing and social distancing, was associated with a decrease in the incidence of RSV and influenza during the pandemic. This suggests that public health measures can effectively reduce the transmission of respiratory viruses, providing a framework for future interventions during outbreaks. The study demonstrates that during the pandemic period, the detection of respiratory pathogens such as SARS-CoV-2 and RV/EV was not only critical for accurate diagnosis but also served as an important indicator of the broader epidemiological trends in respiratory infections. This information is vital for public health systems to maintain vigilance and adapt diagnostic strategies to swiftly identify and treat respiratory infections. The detection of multiple coinfections, including double, triple, and even quadruple infections, highlights the complexity of respiratory infections. Understanding these patterns is essential for clinicians to tailor treatment plans and manage patient outcomes more effectively. We suggest that it will be important for public health to continue to monitor the viral agents that cause RTIs using syndromic testing in the post-pandemic period and to determine how their prevalence changes after the measures are lifted.

## Figures and Tables

**Table 1:** The distribution of the detected pathogens by age groups.

Infections	0–5 (*n* = 1360)	6–18 (*n* = 494)	19–45 (*n* = 68)	>46 (*n* = 62)	Total (*n* = 1984)
Adenovirus	2.35% (*n* = 32)	1.62% (*n* = 8)	0% (*n* = 0)	1.61% (*n* = 1)	2.06% (*n* = 41)
Bocavirus	2.79% (*n* = 38)	0.40% (*n* = 2)	0% (*n* = 0)	0% (*n* = 0)	2.01% (*n* = 40)
Bocavirus/Rhinovirus-Enterovirus	1.03% (*n* = 14)	0% (*n* = 0)	0% (*n* = 0)	0% (*n* = 0)	0.70% (*n* = 14)
Coronavirus 229e	0.44% (*n* = 6)	0.40% (*n* = 2)	2.94% (*n* = 2)	4.84% (*n* = 3)	0.65% (*n* = 13)
HMPV A/B	2.57% (*n* = 35)	1.01% (*n* = 5)	1.47% (*n* = 1)	3.23% (*n* = 2)	2.16% (*n* = 43)
Influenza A	3.38% (*n* = 46)	11.13% (*n* = 55)	10.29% (*n* = 7)	4.84% (*n* = 3)	5.60% (*n* = 111)
Influenza B	0.37% (*n* = 5)	1.21% (*n* = 6)	0% (*n* = 0)	0% (*n* = 0)	0.55% (*n* = 11)
Rhinovirus-Enterovirus	21.03% (*n* = 286)	14.37% (*n* = 71)	13.24% (*n* = 9)	4.84% (*n* = 3)	18.60% (*n* = 369)
Rhinovirus-Enterovirus/RSV A/B	1.91% (*n* = 26)	0.40% (*n* = 2)	0% (*n* = 0)	0% (*n* = 0)	1.41% (*n* = 28)
RSV A/B	10.44% (*n* = 142)	2.23% (*n* = 11)	1.47% (*n* = 1)	4.84% (*n* = 3)	7.91% (*n* = 157)
SARS-CoV-2	3.82% (*n* = 52)	5.67% (*n* = 28)	22.06% (*n* = 15)	8.06% (*n* = 5)	5.04% (*n* = 100)
Total Positive	62.50% (*n* = 850)	44.13% (*n* = 218)	51.47% (*n* = 35)	37.10% (*n* = 23)	56.75% (*n* = 1126)
Total Negative	37.50% (*n* = 510)	55.87% (*n* = 276)	48.53% (*n* = 33)	62.90% (*n* = 39)	43.25% (*n* = 858)

SARS-CoV-2: severe acute respiratory syndrome virus 2, RSV A/B: respiratory syncytial virus A/B; HMPV A/B: human metapneumovirus A/B.

**Table 2:** Distribution of detected pathogens by years (*n*).

Infections	2021 (*n* = 710)	2022 (*n* = 240)	2023 (*n* = 759)
SARS-CoV-2	16 (2.25%)	50 (20.83%)	34 (4.47%)
RSV A/B	90 (12.67%)	7 (2.91%)	60 (7.90%)
Rhinovirus/Enterovirus	151 (21.26%)	36 (15%)	182 (23.97%)
Rhinovirus/Enterovirus/RSV A/B	12 (1.69%)	6 (2.50%)	10 (1.31%)
Influenza A	78 (10.98%)	29 (12.08%)	34 (4.47%)
Influenza B	0 (0%)	0 (0%)	11 (1.44%)
HMPV A/B	22 (3.10%)	32 (13.33%)	13 (1.71%)
Bocavirus	20 (2.81%)	0 (0%)	20 (2.63%)
Bocavirus/Rhinovirus/Enterovirus	6 (0.84%)	5 (2.08%)	3 (0.39%)
Adenovirus	20 (2.81%)	8 (3.33%)	13 (1.71%)
Coronavirus 229E	5 (0.70%)	6 (2.50%)	2 (0.26%)

SARS-CoV-2: severe acute respiratory syndrome coronavirus 2, RSV A/B: respiratory syncytial virus A/B; HMPV A/B: human metapneumovirus A/B.

**Table 3:** The distribution of the mostly detected pathogens according to three-month periods.

Pathogens	1st Trimester *	2nd Trimester *	3rd Trimester *	4th Trimester *
Rhinovirus/Enterovirus	29.39	53.17	51.96	58.59
Rhinovirus/Enterovirus/SARS-CoV-2	42.74	1.58	0.74	1.02

* 1st Trimester (January, February, March), 2nd Trimester (April, May, June), 3rd Trimester (July, August, September); 4th Trimester (October, November, December). SARS-CoV-2: severe acute respiratory syndrome coronavirus 2.

**Table 4:** The significance and standard deviation values by months.

Months	N *	Mean ± SD	*p*
January	75	0.924 ± 2.688	<0.001
February	75	0.820 ± 3.471	0.002
March	75	0.720 ± 4.193	0.006
April	75	0.567 ± 2.242	0.003
May	75	0.662 ± 2.509	0.001
June	75	0.732 ± 2.594	<0.001
July	75	0.570 ± 2.043	<0.001
August	75	0.481 ± 1.588	<0.001
September	75	0.687 ± 3.303	<0.001
October	75	0.811 ± 3.505	<0.001
November	75	0.852 ± 2.799	<0.001
December	75	0.905 ± 3.001	<0.001

* “N” refers to the 75 different infection combinations detected in the present study.

## Data Availability

The data presented in this study are available on request from the corresponding author.

## Abbreviations

The following abbreviations are used in this manuscript:

RV/EV	Rhinovirus/Enterovirus
IAV	Influenza A
IBV	Influenza B
RSV	Respiratory syncytial virus
hMPV	Human metapneumovirus
hADV	Human adenovirus
PIV	Parainfluenza virus
RV	Rhinovirus
